# Outcomes and costs of blunt trauma in England and Wales

**DOI:** 10.1186/cc6797

**Published:** 2008-02-19

**Authors:** Michael C Christensen, Saxon Ridley, Fiona E Lecky, Vicki Munro, Stephen Morris

**Affiliations:** 1 Global Development, Novo Nordisk A/S, Krogshøjvej 55, DK-2880 Bagsværd, Denmark; 2 Glan Clwyd Hospital, Rhyl LL18 5UJ, UK; 3 Trauma Audit and Research Network, University of Manchester, Eccles Old Road, Salford M6 8HD, UK; 4 Novo Nordisk Ltd, Broadfield Park, Brighton Road, Crawley RH11 9RT, UK; 5 Health Economics Research Group, Brunel University, Uxbridge UB8 3PH, UK

## Abstract

**Background:**

Trauma represents an important public health concern in the United Kingdom, yet the acute costs of blunt trauma injury have not been documented and analysed in detail. Knowledge of the overall costs of trauma care, and the drivers of these costs, is a prerequisite for a cost-conscious approach to improvement in standards of trauma care, including evaluation of the cost-effectiveness of new healthcare technologies.

**Methods:**

Using the Trauma Audit Research Network database, we examined patient records for persons aged 18 years and older hospitalised for blunt trauma between January 2000 and December 2005. Patients were stratified by the Injury Severity Score (ISS).

**Results:**

A total of 35,564 patients were identified; 60% with an ISS of 0 to 9, 17% with an ISS of 10 to 16, 12% with an ISS of 17 to 25, and 11% with an ISS of 26 to 75. The median age was 46 years and 63% of patients were men. Falls were the most common cause of injury (50%), followed by road traffic collisions (33%). Twenty-nine percent of patients were admitted to critical care for a median length of stay of 4 days. The median total hospital length of stay was 9 days, and 69% of patients underwent at least one surgical procedure. Seven percent of the patients died before discharge, with the highest proportion of deaths among those in the ISS 26–75 group (32%). The mean hospital cost per person was £9,530 (± 11,872). Costs varied significantly by Glasgow Coma Score, ISS, age, cause of injury, type of injury, hospital mortality, grade and specialty of doctor seen in the accident and emergency department, and year of admission.

**Conclusion:**

The acute treatment costs of blunt trauma in England and Wales vary significantly by injury severity and survival, and public health initiatives that aim to reduce both the incidence and severity of blunt trauma are likely to produce significant savings in acute trauma care. The largest component of acute hospital cost is determined by the length of stay, and measures designed to reduce length of admissions are likely to be the most effective in reducing the costs of blunt trauma care.

## Introduction

Severe injury remains a common cause of death and permanent disability across all ages in the United Kingdom, and implies substantial costs to society in medical care, premature death, disability and use of social services [1,2]. Injuries are a major cause of morbidity and mortality in young people, representing the leading cause of death in those aged younger than 35 years and causing approximately 3,500 deaths annually in England and Wales [3]. The number of serious injuries implies approximately 640,000 hospitals admissions each year and more than 6 million attendances to accident and emergency (A&E) departments [4,5].

The incidence of polytrauma (defined as an injury severity score (ISS) > 15) is currently 10,500 per year, equivalent to one patient per 1,000 presenting in A&E departments [6]. While the number of people killed or severely injured in a road traffic collision (RTC) has declined significantly over the past four decades, the numbers are still substantial: in 2005 there were 2,913 fatal road accidents, 25,029 serious road accidents and 170,793 minor road accidents in the United Kingdom [7]. The value to society of preventing these accidents was estimated at £12,807 million, or £64,440 per accident [8]. The annual cost to the National Health Service (NHS) of treating trauma injuries is currently estimated at £1.6 billion; about 7% of the total annual NHS budget [2].

In light of the clinical and economic consequences of severe injury, the Royal College of Surgeons and the British Orthopaedic Association recommend developing a national system of trauma services based on the geographic trauma systems for England, Wales and Northern Ireland [1]. In addition, they recommend specific standards in trauma care for initial assessment and resuscitation, anaesthesia, intensive care and rehabilitation to provide hospital trusts with reasonable goals to which they could audit current clinical practice. Both organisations argue that improvement of care for the severely injured will create an opportunity for reducing the costs of avoidable death and unnecessary morbidity.

Recommendations for improving standards of care are likely to require additional funding and resources, raising questions about the cost-effectiveness of such healthcare improvements. While the outcomes of severe injury have been well documented over the past two decades and some information exists on the standards of trauma care, there are little data on the medical costs of severe injury in the United Kingdom. In particular, there is no information on the major determinants of hospital costs; for example, types of injuries and patient characteristics that drive the total costs of trauma care. Such information would usefully contribute to policy decision-making by targeting high-cost components in trauma care. It would also be a useful resource for evaluating the cost-effectiveness of trauma care interventions.

The aim of the present paper is to analyse the determinants of the costs of trauma care in England and Wales. We examine recent data on acute hospital treatment for blunt trauma and we estimate the acute hospital costs from the perspective of the NHS. We also identify the most important predictors of acute care costs in terms of patient and injury characteristics.

## Methods

### Data and variables

Data for this study were obtained from the Trauma Audit Research Network (TARN). Data from TARN have been used to examine a range of topics in trauma and trauma care, including trends in trauma care [9], the effect of neurosurgical care on head injury outcome [10], and outcome prediction in trauma [11]. The present study is the first to use TARN data to investigate the acute treatment costs of blunt trauma patients.

The TARN registry includes 50% of all hospitals receiving trauma patients in England and Wales [12]. The registry collects data on patients who sustain injuries resulting in immediate admission to hospital for ≥ 3 days, admission to an intensive care unit or a high dependency unit, or death within 93 days. Patients over 65 years old with isolated fracture of the neck of femur/pubic ramus and those with simple isolated injuries are excluded.

Data for this study came exclusively from the TARN database without author access to patient records. The TARN database is supported by the Healthcare Commission. Specific informed patient consent or ethical approval is not required because no patient identifiers are retained by TARN electronically or on paper. The TARN has Patient Information Advisory Group approval.

We included all patients hospitalised for blunt trauma in TARN hospitals between 1 January 2000 and 31 December 2005. Each hospital completes for every patient a data entry sheet with information on age, gender, cause and type of injuries, severity of injuries, treatment provided at the scene of incident, treatment en route to hospital, treatment in the A&E department, and any other care received at the hospital, including diagnostic tests, surgical procedures, and length of stay. Records of patients transferred between hospitals are linked so that full data for these patients are available.

In our study, injury severity is measured using the Injury Severity Score (ISS) [13]. The ISS is based on an ordinal scale ranging from 0 to 75, which is in turn based on the Abbreviated Injury Scale (AIS). Each AIS injury code is allocated an AIS score ranging from 1 (minor injury) to 6 (virtually unsurvivable injury). To compute the ISS, AIS injury codes are grouped into six body regions (head or neck, face, chest, abdominal or pelvic, extremities, and external) and the ISS is calculated as the sum of the squares of the highest AIS scores for the three most severely injured body regions. AIS codes of 6 are automatically allocated an ISS of 75. As no definitive grouping of ISS scores exists [14], we used the following categories: ISS 0–9 group, ISS 10–16 group, ISS 17–25 group and ISS 26–75 group. This stratification allows one injury with an AIS score of 3 in the first category, one injury with an AIS score of 4 in the second category, and one injury with an AIS score of 5 in the third category. In addition, we used data on gender, age, cause of injury, earliest recorded Glasgow Coma Score (GCS), type and number of injuries sustained, discharge status, and year of admission.

We also used data from the TARN registry on the characteristics of the treatment received. These data included the mode of arrival at the hospital, the time from the emergency call to arrival at the A&E department (when by ambulance), the highest grade and specialty of the doctor attending the patient in the A&E department, the time spent in the A&E department, surgery, admission to critical care, and the length of stay. We also used data on whether or not the treating hospital had a neurosurgical centre and/or a spinal injury unit as a rough indicator of specialisation in trauma care. We excluded from the analysis patients younger than 18 years of age at the time of injury.

### Measuring costs

To estimate costs we took the perspective of the NHS in England and Wales; that is, only costs to the NHS in these regions were considered. We focused on the costs of the treatment received during the initial acute hospitalisation. We calculated treatment costs for each patient based on the following cost components: cost of transportation (ambulance, helicopter), cost of hospital stay (A&E department, critical care, general ward), and cost of all surgical procedures performed. Resource use for every component was recorded for each patient in the TARN database using the data entry sheet. For all resource use we assigned unit costs obtained from external sources to each item (see Table 1). All costs were measured in 2004 UK sterling.

**Table 1:** Unit costs

Cost component	Unit or duration (min)	Unit cost (£)	Source and notes
Mode of arrival at hospital			
Ambulance	Cost per minute	5.50	Curtis and Netten (p. 112) [16]
			Cost per minute of emergency ambulance service
Helicopter	Mean cost per patient journey	1,650	London Air Ambulance website
			Mean cost per mission
Hospital stay			
Accident and emergency department	Mean cost per attender	278	Department of Health [17]
			Mean cost per attender across all accident and emergency Healthcare Resource Groups
General ward	Mean cost per day	281	Department of Health [17]
			Mean national average unit cost per day across all nonelective Healthcare Resource Groups
Critical care unit	Mean cost per day	1,328	Department of Health [17]
			Mean cost per day in intensive care unit/intensive therapy unit
Surgical procedures^a^			
Open reduction and internal fixation	126	1,597	Trauma Audit Research Network, National Institute for Health and Clinical Excellence [18]
Nail insertion	168	1,861	
Wound debridement	139	1,680	
Manipulation of bone	69	1,230	
Screw insertion	111	1,499	
External fixation	147	1,731	
Evacuation of extradural haematoma/subdural haematoma	138	1,673	
K-wire insertion	102	1,443	
Intercranial pressure monitor	97	1,410	
Craniotomy	175	1,906	

^a^Details reported for the 10 most common procedures only; 103 procedures are included in the analysis. The duration and unit cost range from 10 minutes and £852 for gastroscopy to 475 minutes and £3,828 for escharatomy. The duration (min) for each procedure was computed internally using the Trauma Audit Research Network database. The unit costs were then computed by multiplying the duration by the variable cost per minute from National Institute for Health and Clinical Excellence and adding a fixed cost per procedure also taken from National Institute for Health and Clinical Excellence [18]. This method has been used in previous UK cost analyses of trauma care [19]>.

### Analysis

We first examined the demographic and clinical characteristics of the study sample and the acute treatment provided across groups of patients stratified by ISS. We then computed treatment costs for every patient and calculated the mean treatment costs by patient, by injury and treatment characteristics and by ISS group.

To identify significant independent determinants of acute treatment costs we undertook multivariate regression analysis. We regressed individual-level treatment costs against patient, injury and treatment characteristics. We excluded the variables used in the construction of the treatment cost variable. The variables included in the final regression model were selected using forward stepwise and backward stepwise selection procedures, where the significance level for removal from the model was *P *≥ 0.01 and the significance level for addition to the model was *P *< 0.005. We also included 43 hospital indicators denoting the hospital at which the patient was treated. By including these indicators we aimed to control for variations in costs that are due to variations in provider practice rather than due to injury type, severity, and general treatment patterns. We report robust standard errors that control for clustering within individuals. The model is estimated using least squares and the coefficients are marginal effects.

Some of the data collected by the TARN registry and used in the present analysis are missing in a number of patients. The main variables with missing information are the time from emergency call to arrival at the A&E department and the surgical procedures. The latter may be missing because the relevant section on the data entry sheet completed by TARN participants describing the operative procedures is completed by free text and may have been left blank. We report the numbers of observations used to calculate every statistic reported. When we calculated the acute care costs for each patient, we did so using only patients with complete data. If data for one or more of the cost components were missing for a patient we assigned a 'missing' code to the total costs incurred by that patient, and we did not include the patient in the cost calculation.

## Results

### Patient and injury characteristics

We identified 35,564 patients with blunt injuries (blunt injury, nonpenetrating trauma or blunt force trauma) in the study period (Table 2). Sixty percent had an ISS of 0 to 9 (21,280 patients), 17% had an ISS of 10 to 16 (6,075 patients), 12% had an ISS of 17 to 25 (4,375 patients), and 11% had an ISS of 26 to 75 (3,834 patients). Sixty-three percent of the sample was male, and the median age was 46 years (interquartile range 31 to 63 years). There was a higher than average proportion of males in the ISS 10–16 group, the ISS 17–25 group and the ISS 26–75 group, and the median age was lower in these groups.

**Table 2:** Patient and injury characteristics

	Injury Severity Score				
	
	0–9	10–16	17–25	26–75	All
Total number of patients	21,280	6,075	4,375	3,834	35,564
Gender (%)					
Male	55.5	69.7	75.2	76.9	62.6
Female	44.6	30.3	24.9	23.1	37.4
Age (years)					
Median (interquartile range)	49 (33 to 66)	43 (29 to 60)	42 (29 to 58)	39 (27 to 56)	46 (31 to 63)
Cause of injury (%)					
Road traffic collisions	20.0	47.1	46.8	62.8	32.5
Fall more than 2 metres	10.7	14.9	19.6	18.3	13.3
Fall less than 2 metres	51.6	20.5	14.1	7.2	36.9
Blow(s)	4.6	9.7	9.9	4.8	6.1
Other	13.2	7.7	9.7	6.9	11.2
Glasgow Coma Score					
Median (interquartile range)	15 (15 to 15)	15 (15 to 15)	15 (10 to 15)	10 (4 to 15)	15 (15 to 15)
*n*	19,026	5,651	3,790	3,290	31,757
Serious injuries by body region (%)^a^					
Head	1.7	24.0	55.2	72.1	19.7
Face	0.2	1.2	2.9	6.3	1.3
Thorax	1.8	12.5	28.1	54.7	12.6
Abdomen	0.2	1.3	5.1	16.0	2.7
Spine	2.0	6.4	9.6	12.3	4.8
Upper limb	18.1	21.0	11.5	16.8	17.7
Lower limb	46.4	36.0	21.7	34.5	40.3
External/burn	0.8	2.1	3.7	2.3	1.6
Mean number of serious injuries^a^	0.8	1.5	2.5	4.4	1.5
Inhospital mortality (%)	2.0	4.3	14.8	32.3	7.3

Sample size is equal to the total number other than where indicated by *n*.

^a^Injuries with an Abbreviated Injury Scale score ≥ 3.

Across all patients the most common cause of injury was falls (50%), followed by RTC (33%); RTC was the most common cause of injury among patients with an ISS of 10 to 75. The median Glasgow Coma Score was 15 (interquartile range 15 to 15) across the whole sample, but was only 10 (interquartile range 4 to 15) in the ISS 26–75 group. The most common serious injuries (with AIS ≥ 3) were injuries to the lower limbs (40%), the head (20%), the upper limbs (18%) and the chest (13%). The average patient sustained 1.5 injuries with an AIS score ≥ 3, ranging from 0.8 in the ISS 0–9 group to 4.4 in the ISS 26–75 group. Seven percent of patients died in hospital, with the highest proportion of deaths among those in the ISS 26–75 group (32%). Of the hospital deaths observed, the mean (standard deviation (SD)) time to death was 9.3 days (20.3 days) from the date of arrival at the hospital. The time to death was 7.5 days (21.7 days) for RTC, 7.6 days (17.1 days) for falls more than 2 metres, 14.4 days (19.7 days) for falls less than 2 meters, and 5.6 days (9.3 days) for blows.

### Treatment characteristics

Eighty percent of the patients arrived at the hospital by ambulance, 11% by car, and 6% by helicopter (Table 3). The mean (SD) time from emergency call to arrival at the A&E department was 71 minutes (95 min), and the mean (SD) time in the A&E department was 194 minutes (155 min). Fifteen percent of patients were seen by a consultant in the A&E department, 42% were seen by a middle-grade doctor and 39% were seen by a senior house officer. The proportion seeing a consultant was highest (32%) in the most severely injured group of patients (ISS 26–75 group). Seventy-four percent of patients were seen by a doctor specialising in emergency medicine; attending specialists in orthopaedics (42%), anaesthesia (13%), neurosurgery (10%), and general surgery (9%) were also common.

**Table 3:** Treatment characteristics

	Injury Severity Score				
	
	0–9	10–16	17–25	26–75	All
Mode of arrival (%)					
Ambulance	77.3	85.9	86.4	80.4	80.2
Helicopter	2.9	7.6	10.6	17.7	6.2
Car	15.9	4.9	1.7	0.4	10.6
Walking	1.1	0.4	0.2	0.1	0.8
Other	2.9	1.2	1.2	1.4	2.3
*n*	19,713	5,625	3,875	3,559	32,772
Time from emergency call to arrival at the A&E department (minutes)^a^					
Mean (standard deviation)	59.6 (64.8)	70.6 (94.8)	100.9 (149.5)	100.5 (136.3)	70.7 (94.7)
*n*	7,493	2,247	1,482	1,307	12,529
Time in the A&E department (minutes)					
Mean (standard deviation)	217.0 (144.7)	195.9 (166.0)	149.6 (170.4)	120.5 (141.5)	193.8 (155.4)
*n*	14,262	4,154	3,020	2,827	24,263
Grade of most senior doctor seen in the A&E department (%)					
Consultant	9.2	18.0	24.1	32.4	14.9
Middle grade	35.0	49.3	57.6	56.4	42.3
Senior house officer	50.9	30.1	16.1	9.3	38.9
*n*	20,078	5,592	3,895	3,480	33,045
Specialty of doctors seen in the A&E department (%)^b^					
Emergency medicine	83.4	68.2	53.8	50.7	73.8
Orthopaedics	48.7	39.0	27.0	29.5	42.4
Anaesthesia	4.0	16.7	30.8	41.5	13.3
Neurosurgery	2.5	12.8	24.2	27.0	9.5
General surgery	3.9	12.5	16.6	21.9	8.8
Other	9.2	15.7	22.0	22.8	13.3
*n*	20,263	5,651	3,994	3,588	33,496
Surgery (%)^c^					
Bone fixation^d^	60.9	38.5	19.4	18.5	47.4
Wound debridement	5.6	8.4	5.2	5.0	6.0
Intracranial procedures^e^	0.2	5.3	18.2	23.6	5.8
*n*	21,280	6,075	4,375	3,834	35,564
Admitted to critical care					
Percentage	9.6	31.3	61.2	79.1	29.0
*n*	13,427	4,374	3,256	2,895	23,952
Length of stay in critical care (days)^f^					
Median (interquartile range)	2 (1 to 5)	3 (2 to 7)	4 (2 to 10)	7 (3 to 15)	4 (2 to 10)
*n*	1,287	1,368	1,991	2,290	6,936
Total length of stay (days)^g^					
Median (interquartile range)	8 (5 to 14)	10 (5 to 19)	13 (6 to 26)	17 (6 to 39)	9 (5 to 19)
*n*	13,427	4,374	3,256	2,895	23,952
Hospital has a neurosurgical centre					
Percentage	46.9	54.4	61.2	67.4	52.2
*n*	21,280	6,075	4,375	3,834	35,564
Hospital has a spinal injuries unit					
Percentage	6.2	5.3	3.9	3.6	5.5
*n*	21,280	6,075	4,375	3,834	35,564

^a^Among those arriving by ambulance at the accident and emergency (A&E) department. ^b^Patients could be seen by doctors from more than one specialty; hence the numbers sum to more than 100%. ^c^Based on the 10 most common procedures. ^d^Including open reduction and internal fixation, nail insertion, manipulation of bone, screw insertion, external fixation and K-wire insertion. ^e^Including evacuation of extradural haematoma/subdural haematoma, intracranial pressure monitor and craniotomy. ^f^Figures pertain only to those patients who were admitted to critical care. ^g^Including length of stay in critical care; figures pertain both to those who were admitted to critical care and those who were not.

Sixty nine percent of patients underwent at least one surgical procedure; the most common were bone fixation (47%), wound debridement (6%) and intracranial procedures (6%). Open reduction and internal fixation was the most common procedure in the ISS 0–9 group, the ISS 10–16 group and the ISS 17–25 group (35%, 19% and 10% of patients, respectively). Evacuation of extradural haematoma/subdural haematoma was the most common procedure in the ISS 26–75 group (9%).

Twenty-nine percent of patients were admitted to critical care. The percentage ranged from 10% in the ISS 0–9 group to 79% in the ISS 26–75 group. It is noteworthy that the percentage of patients in the ISS 26–75 group admitted to critical care was less than 100%. This finding may be explained by an insufficient number of critical care beds available to meet the demand. The median length of stay in critical care among those who were admitted was 4 days (interquartile range 2 to 10 days). As expected, the median length of stay in critical care was shortest in the ISS 0–9 group (2 days, interquartile range 1 to 5 days) and longest in the ISS 26–75 group (7 days, interquartile range 3 to 15 days). The median total length of stay, including the length of stay on a general ward and in critical care, was 9 days (interquartile range 5 to 19 days) across the whole sample – ranging from 8 days (interquartile range 5 to 14 days) in the ISS 0–9 group to 17 days (interquartile range 6 to 39 days) in the ISS 26–75 group.

Fifty-two percent of patients were treated in a hospital with a neurosurgical centre, and 6% of patients were treated in a hospital with a spinal injuries unit.

### Treatment costs

The mean (SD) total hospital cost per patient was £9,530 (± 11,872) (Table 4). Across all patients, the length of stay in a general ward accounted for the largest percentage of costs (46%), followed by the length of stay in critical care (29%). The next most important component was surgical procedures (15%), followed by travel to the A&E department (5%) and A&E costs (4%). These proportions were not the same for every ISS group: for patients in the ISS 17–25 and the ISS 26–75 groups the length of stay in critical care was the largest component of cost, accounting for 42% and 52% of the mean total costs, respectively. Mean costs increased with ISS up to a mean cost of £21,173 in the ISS 26–75 group.

**Table 4:** Mean treatment costs by patient and injury characteristics

Cost (£)	Injury Severity Score				
	
	0–9	10–16	17–25	26–75	All
**Total cost**					
Mean (standard deviation)	6,198 (5,782)	8,989 (10,276)	14,205 (14,910)	21,173 (20,177)	9,530 (11,872)
Median (interquartile range)	4,668 (3,475 to 6,861)	5,692 (3,632 to 9,986)	8,884 (4,660 to 18,444)	15,041 (6,205 to 30,266)	5,390 (3,626 to 9815)
*n*	12,775	4,105	2,910	2,678	22,468
**Mean total cost**					
By gender					
Male	6,081	9,041	14,128	21,283	10,158
Female	6,343	8,865	14,454	20,786	8,433
By age group					
18 to 24 years	5,506	8,053	12,808	19,495	9,563
25 to 44 years	5,507	8,431	13,379	22,176	9,588
45 to 64 years	6,035	9,877	15,236	23,815	9,663
65 to 74 years	6,947	10,879	18,305	16,936	9,531
75+ years	7,920	9,117	14,237	15,171	9,050
By cause of injury					
Road traffic collisions	7,244	9,686	15,485	22,950	12,817
Fall more than 2 metres	5,980	9,639	13,769	19,852	10,351
Fall less than 2 metres	6,015	7,585	12,534	13,161	6,637
Blow(s)	5,055	6,742	10,756	14,548	7,791
Other	5,799	9,967	14,513	21,232	8,378
By Glasgow Coma Score group					
3 to 5	14,695	17,592	17,929	21,832	19,891
6 to 8	14,376	17,461	18,953	24,686	20,865
9 to 12	9,064	10,809	14,623	22,157	15,800
13 to 15	6,072	7,972	12,291	18,823	7,837
By serious injuries by body region^a^					
Head	8,426	10,243	13,762	20,532	15,555
Face	7,403	7,589	11,881	21,363	16,105
Thorax	5,291	7,763	14,434	23,835	16,932
Abdomen	7,062	10,341	15,760	24,075	19,983
Spine	7,129	8,504	16,213	27,061	15,444
Upper limb	6,108	8,192	16,302	25,534	9,885
Lower limb	6,973	9,740	16,637	25,557	10,084
External/burn	8,677	12,796	17,456	16,713	13,190
By number of serious injuries^a^					
0	5,301	10,466			5,376
1	6,178	7,668	11,632	13,358	6,991
2	8,368	10,019	12,169	16,419	10,832
3	11,210	13,047	15,490	18,721	15,588
4	15,869	15,470	18,729	23,095	20,515
5 or more	18,644	18,565	19,942	24,057	23,027
By discharge status					
Alive	6,129	8,872	14,423	24,268	9,457
Dead	11,675	13,608	11,770	9,978	10,966

^a^Injuries with an Abbreviated Injury Scale score ≥ 3.

There was little impact of gender on costs, but costs increased with age in the ISS 0–9 group and the ISS 10–16 group, and decreased in the elderly in the other ISS groups; this age effect is possibly associated with a higher mortality in the elderly. Treatment costs were highest among patients injured in a RTC, and among patients in coma upon arrival at the hospital (Glasgow Coma Score ≤ 8). Every type of serious injury was associated with higher treatment costs; limb injuries had the smallest additional impact on cost, and abdominal injuries had the largest additional impact. Treatment costs increased with the number of serious injuries sustained by the patient.

In the ISS 0–9 group and the ISS 10–16 group, mortality was associated with higher treatment costs compared with being alive at discharge. Conversely, mortality was associated with lower treatment costs in the ISS 17–25 group and the ISS 26–75 group. Across all patients, mortality implied a slight increase in treatment costs.

The impact of treatment patterns on treatment costs are presented in Table 5. Treatment costs varied by mode of hospital arrival (helicopter is the most expensive, followed by ambulance) and the time from emergency call to arrival at the A&E department (both positively correlated), and by the time in the A&E department (negatively correlated). Mean treatment costs were higher if the patient was seen by a consultant, and if the patient was seen by a doctor from the specialties of anaesthesia, neurosurgery and general surgery. Since the unit costs used in the analysis do not vary by the grade or specialty of the doctors seen in the A&E department, the observed variation in costs by the type of doctor relate to variations in surgery and length of stay, which in turn are affected by injury severity. Patients who were seen by a consultant had more severe injuries than those who were seen by less senior medical personnel, which probably explains the higher costs incurred by this group (mean ISS 17.8 versus 12.0, mean difference 5.8, 95% confidence interval 5.5 to 6.2). Patients treated by doctors from anaesthesia, neurosurgery and general surgery were also more severely injured than those who did not see doctors from these specialties (mean ISS 20.6 versus 10.5, mean difference 10.1, 95% confidence interval 9.8 to 10.3).

**Table 5:** Mean treatment costs by treatment characteristics

Mean total cost (£)	Injury Severity Score				
	
	0–9	10–16	17–25	26–75	All
By mode of arrival					
Ambulance	6,521	9,044	13,774	19,824	9,575
Helicopter	8,939	11,465	18,006	26,608	16,692
Car	4,266	4,580	6,392	5,602	4,346
By time from emergency call to arrival at the A&E department^a^					
0 to 1 hours	6,267	8,635	13,243	17,507	8,651
1 to 2 hours	6,680	8,899	14,482	19,982	9,405
2 to 3 hours	8,618	12,693	10,931	29,073	13,083
3 to 4 hours	12,680	16,074	18,024	26,440	19,286
4+ hours	12,087	14,780	16,875	23,601	17,537
By time in the A&E department					
0 to 1 hours	7,917	11,566	16,465	21,805	14,254
1 to 2 hours	6,512	10,686	18,447	23,575	11,122
2 to 3 hours	6,246	8,833	15,109	22,215	8,984
3 to 4 hours	5,730	7,834	14,139	20,648	8,097
4+ hours	5,898	7,896	12,198	19,172	8,038
By grade of most senior doctor seen in the A&E department					
Consultant	7,283	10,290	15,562	22,261	12,974
Middle grade	6,508	9,350	14,185	20,983	10,524
Senior house officer	5,755	7,227	11,453	16,135	6,585
By specialty of doctors seen in the A&E department					
Emergency medicine	5,906	8,009	13,138	19,844	8,144
Orthopaedics	6,195	9,154	14,805	23,605	8,912
Anaesthesia	10,091	13,162	18,272	23,820	17,697
Neurosurgery	7,858	10,396	14,361	20,906	14,336
General surgery	8,033	10,346	15,939	23,880	14,877
By surgery^b^					
Bone fixation^c^					
No	6,076	9,032	13,577	19,986	10,469
Yes	6,290	8,909	16,793	26,135	8,323
Wound debridement					
No	5,998	8,761	14,045	20,726	9,308
Yes	9,149	11,302	16,683	27,938	12,592
Intracranial procedures^d^					
No	6,169	8,418	13,311	20,678	8,877
Yes	22,430	21,006	19,535	23,157	21,495
By length of stay in critical care					
0 days	5,436	5,628	5,711	5,177	5,480
1 to 5 days	9,514	9,962	10,357	9,866	9,936
6 to 10 days	18,914	20,906	20,086	21,624	20,716
11+ days	42,767	41,730	41,827	45,135	43,589
By total length of stay^e^					
0 to 5 days	3,270	3,142	3,611	3,958	3,350
6 to 10 days	4,544	5,116	6,538	9,074	5,116
11 to 20 days	6,658	8,346	10,858	15,136	8,420
21 to 30 days	10,358	13,472	18,700	21,797	14,344
31 to 40 days	13,830	17,890	23,734	30,116	20,142
41 to 50 days	15,949	22,869	29,074	36,596	25,175
51+ days	26,791	34,697	41,630	49,338	39,025

A&E, accident and emergency. ^a^Among those arriving by ambulance. ^b^Based on the 10 most common procedures. ^c^Including open reduction and internal fixation, nail insertion, manipulation of bone, screw insertion, external fixation and K-wire insertion. ^d^Including evacuation of extradural haematoma/subdural haematoma, intracranial pressure monitor and craniotomy. ^e^Including length of stay in critical care; figures pertain both to those who were admitted to critical care and those who were not.

Surgery for bone fixation was associated with higher treatment costs among patients in some, but not all, ISS groups. Wound debridement and intracranial procedures were associated with higher mean costs in all ISS groups.

As expected, treatment costs were positively correlated with the length of stay – both for critical care length of stay and for total length of stay. Mean treatment costs did not vary by whether or not the hospital in which the patient was treated had either a neurosurgical centre or a spinal injuries unit.

### Multivariate analysis

The results of the multivariate regression analysis of costs are presented in Table 6. The covariates explain 28% of the variation in hospital costs. The most significant determinants of costs were ISS and age (both positively correlated with costs), RTC (significantly higher costs than other causes), and Glasgow Coma Score (negatively correlated with costs, indicating greater severity was associated with higher costs). Serious injuries of the abdomen, thorax, spine and upper and lower limbs were significantly associated with higher treatment costs, while hospital mortality was significantly associated with lower treatment costs. Being seen in the A&E department by a consultant or middle-grade doctor was associated with significantly higher costs than being seen by a senior house officer, and being seen by a doctor from the specialties of anaesthesia or general surgery was also associated with a significant increase in treatment cost. Treatment costs were significantly lower in 2004 and 2005 compared with 2000 to 2003.

**Table 6:** Multivariate analysis of treatment costs

	Coefficient	*t *statistic	*P *value
Injury Severity Scale^a^			
10 to 16	1,062.3	5.8	<0.001
17 to 25	4,328.1	13.5	<0.001
26 to 75	8,921.9	18.4	<0.001
Age group^b^			
25 to 44 years	1,188.3	5.8	<0.001
45 to 64 years	2,777.8	11.9	<0.001
65 to 74 years	4,080.4	12.4	<0.001
75+ years	5,109.6	17.6	<0.001
Cause of injury^c^			
Fall more than 2 metres	-795.4	-3.3	0.001
Fall less than 2 metres	-1,751.8	-9.9	<0.001
Blow(s)	-1,700.0	-6.0	<0.001
Glasgow Coma Score^d^			
9 to 12	-4,863.9	-6.90	<0.001
13 to 15	-8,819.9	-16.5	<0.001
Serious injuries by body region^e^			
Abdomen	3,656.3	4.5	<0.001
Thorax	1,706.0	5.0	<0.001
Spine	4,512.9	7.9	<0.001
Upper limb	1,406.4	7.0	<0.001
Lower limb	3,220.3	20.5	<0.001
Discharge status: dead	-8,678.1	-12.8	<0.001
Grade of most senior doctor seen in the A&E department^f^			
Consultant	1,841.5	7.3	<0.001
Middle grade	518.7	3.8	<0.001
Specialty of doctors seen in the A&E department			
Anaesthesia	2,476.1	6.3	<0.001
General surgery	1,409.6	3.8	<0.001
Year of admission^g^			
2004	-633.4	-3.3	0.001
2005	-1,152.7	-5.1	<0.001
Observations	19,441		
Adjusted *R*^2^	0.283		

Model also includes 43 hospital indicators. A&E, accident and emergency. ^a^Omitted category is 0 to 9. ^b^Omitted category is 18 to 24 years. ^c^Omitted category is road traffic collision. ^d^Omitted category is 3 to 8. ^e^Injuries with an Abbreviated Injury Scale score ≥ 3. ^f^Omitted category is senior house officer. ^g^Omitted category is 2000 to 2003.

## Discussion

This study provides the first detailed description of the demographic and clinical characteristics of blunt trauma patients in England and Wales, their causes of injury, the acute treatment received and outcomes. The present study is the first to provide a detailed assessment of the NHS hospital costs associated with blunt trauma in England and Wales. The average blunt trauma patient was male, 46 years old, injured by a fall, with an ISS in the range 0 to 9. The most frequent serious injuries were to the upper and lower limbs, head and chest. Around 30% of all blunt trauma patients were admitted to critical care for a median of 4 days. The total median hospital length of stay was 9 days; 7% of the patients died before hospital discharge. The average cost of acute treatment to the NHS was £9,500 per patient.

Few studies have been published on the cost of acute treatment for blunt trauma in the United Kingdom; hence it is difficult to assess the robustness of our cost estimates. In one study, Sikand and colleagues examined the cost implications of treating 69 polytrauma patients (defined as ISS > 15) admitted to an English university hospital in 2000 [15]. The authors reported an average cost of acute care of £14,129 per patient. In our study the costs per patient with ISS > 15 are slightly higher at £16,776; presumably the small difference is explained mainly by changes in NHS pay and prices over time.

Our results suggest that influential determinants of cost were the cause of injury (especially falls and RTCs) and the age of the patient. This influence emphasises the importance of continued public health education and accident prevention measures. From the hospital perspective, the most important cost drivers are length of stay in critical care and total hospital length of stay, accounting for 75% of total costs and far exceeding other components such as transport to hospital, immediate resuscitation in the A&E department, and trauma surgery. In the hospital setting, this suggests that steps to reduce the hospital length of stay (such as expeditious surgery, reduced discharge delays from critical care, outreach services support and earlier placement on a rehabilitation programme) are likely to be more effective, all else equal, than other measures aimed at reducing the costs of blunt trauma care.

The year of admission was a significant predictor of costs, with lower mean treatment costs in the latter part of our study. This may be due to changes in the NHS costs over the period 2000 to 2005, or to improvement in the hospital care pathways introduced to enhance the cost-effectiveness of trauma care; however, the time series is too short to be able to draw any firm conclusions.

We chose to examine the costs of blunt trauma because, in the United Kingdom, this is the largest group of trauma patients – representing 98% of patients in the TARN registry. Any interventions directed at blunt trauma victims or improvements in the care of blunt trauma victims are therefore likely to have the largest public health benefit.

Since 1994 the Department for Transport in England has published annual costs estimates for road casualties for use in cost–benefit analyses of road safety schemes. The latest estimates of the medical and ambulance costs associated with slight, serious and fatal injuries from road traffic collisions in 2005 were £850, £11,460 and £840, respectively [8]. The mean medical and ambulance costs across all casualties were £1,980. These cost estimates represent figures updated from a 1994 study and are significantly lower than the costs observed in our study: the mean costs per person injured in a road traffic collision were £12,800, ranging from £7,200 for the least severely injured to £23,000 for the most severely injured patients. The mean treatment costs in our study among those who died in a RTC were £12,000. If the Department of Transport figures underestimate the true costs of road trauma then they are likely to underestimate the value of potential road safety schemes. The cost estimates generated in our study are likely to be biased upwards because they exclude patients who died before they reached the hospital, and exclude patients hospitalised for less than 3 days and without admission to an intensive care unit/high dependency unit. Both of these patient groups would probably have incurred lower hospital costs than those in our study. Nonetheless, our figures suggest that the cost estimates used by the Department of Transport should be updated.

The mortality rate observed in the current study (7%) suggests that no improvement in survival after blunt trauma has occurred over the past decade in England and Wales. In the most recent assessment among 129,979 blunt trauma patients admitted to hospital between 1989 and 2000, the in-hospital mortality rate was 5.6% among patients with a complete revised trauma score (107,282 patients) and 7.4% among those with an incomplete revised trauma score (46,949 patients) [9]. The authors of the study reported a decline in the severity adjusted odds of death of 3% per year since 1989, although much of the reduction occurred between 1989 and 1994.

We acknowledge a number of limitations to our study. First, we did not include treatment cost occurring after the initial hospitalisation period, including the costs of rehabilitation, home care support and any subsequent hospitalisations related to blunt trauma.

Second, the retrospective nature of the study implies reliance on the quality and completeness of the data reported to the TARN registry. We observed incomplete data on a number of important treatment parameters, particularly the time from emergency call to arrival at the A&E department and surgical procedures. If these data, particularly those relating to surgical procedures, are not missing at random across the recorded patient characteristics, then our results could be biased.

Third, we excluded patients who were admitted for fewer than 3 days and who died before they reached the hospital. Those patients who were not admitted and those who died will have incurred lower treatment costs than those who were admitted, and therefore our estimates are likely to have overestimated the costs of blunt trauma care. Since those who were not admitted are likely to be less severely injured than those patients who were admitted, the extent of the overestimation is likely to be higher for those in the less severely injured ISS groups.

Fourth, the indirect costs related to lost productivity and time spent away from other activities, as well as the costs associated with the pain and suffering by victims and relatives, were not included. The cost of follow-up care and the indirect costs can be substantial, and are likely to represent the majority of the lifetime societal costs of blunt trauma.

Finally, the unit costs used in the analysis are, by necessity, crude. In particular, the unit costs of hospital stays are not specific to blunt trauma patients. Clearly it would have been more appropriate to use the actual costs incurred by each trauma patient included in the analysis, yet these data were not available for our study.

## Conclusion

In this study we provide the first detailed information on treatment patterns, mortality and costs of blunt trauma in the acute care setting in England and Wales. Our findings indicate that the initial hospital costs associated with blunt trauma vary significantly by patient characteristics, by injury characteristics and by treatment characteristics. The largest component of treatment cost, however, is determined by the total hospital length of stay. Measures to reduce the hospital length of stay – either directly in terms of organisation and staffing, or indirectly in terms of better health outcomes – are likely to be the most effective in reducing the costs of blunt trauma care.

## Key messages

• The mean acute hospital cost per blunt trauma patient in England during 2000 to 2005 was £9,530.

• The length of stay on a general ward accounted for the largest percentage of these costs (46%), followed by the length of stay in critical care (29%).

• Acute hospital costs vary significantly by Glasgow Coma Score, ISS, age, cause of injury, body region of injury, hospital mortality, grade and specialty of doctor seen in the A&E department, and year of admission.

## Abbreviations

A&E = accident and emergency; AIS = Abbreviated Injury Scale; ISS = Injury Severity Score; NHS = National Health Service; RTC = road traffic collision; SD = standard deviation; TARN = Trauma Audit Research Network.

## Competing interests

The present study was funded by Novo Nordisk A/S. MCC is an employee of Novo Nordisk A/S, and VM is an employee of Novo Nordisk Ltd. SM has received consultancy fees and SR has received honoraria from Novo Nordisk Ltd. TARN received an unrestricted grant for making data available for the present study.

## Authors' contributions

MCC conceived of the study, participated in study design and coordination, interpreted data, and helped to draft the manuscript. SM participated in the design of the study, performed the statistical analyses, and helped to draft the manuscript. SR and VM participated in the study design and interpretation of data, and helped to draft the manuscript. FEL participated in the study design, the coordination of data collection, and helped to draft the manuscript. All authors read and approved the final manuscript.
